# Impact of the Severe acute respiratory syndrome coronavirus 2 pandemic on mortality associated with healthcare-associated infections

**DOI:** 10.1017/ash.2023.409

**Published:** 2023-08-29

**Authors:** Andrew Atkinson, Katelin B. Nickel, John M. Sahrmann, Dustin Stwalley, Erik R. Dubberke, Kathleen McMullen, Jonas Marschall, Margaret A. Olsen, Jennie H. Kwon, Jason P. Burnham

**Affiliations:** 1 Division of Infectious Diseases, Washington University in St. Louis School of Medicine, St. Louis, MO, USA; 2 Institute for Informatics, Washington University in St. Louis, St. Louis, MO, USA; 3 Infection Prevention, Mercy Hospital, St. Louis, MO, USA

**Keywords:** SARS-CoV-2, COVID-19, healthcare-associated infections

## Abstract

**Objective:**

To determine the relationship between severe acute respiratory syndrome coronavirus 2 infection, hospital-acquired infections (HAIs), and mortality.

**Design:**

Retrospective cohort.

**Setting:**

Three St. Louis, MO hospitals.

**Patients:**

Adults admitted ≥48 hours from January 1, 2017 to August 31, 2020.

**Methods:**

Hospital-acquired infections were defined as those occurring ≥48 hours after admission and were based on positive urine, respiratory, and blood cultures. Poisson interrupted time series compared mortality trajectory before (beginning January 1, 2017) and during the first 6 months of the pandemic. Multivariable logistic regression models were fitted to identify risk factors for mortality in patients with an HAI before and during the pandemic. A time-to-event analysis considered time to death and discharge by fitting Cox proportional hazards models.

**Results:**

Among 6,447 admissions with subsequent HAIs, patients were predominantly White (67.9%), with more females (50.9% vs 46.1%, *P* = .02), having slightly lower body mass index (28 vs 29, *P* = .001), and more having private insurance (50.6% vs 45.7%, *P* = .01) in the pre-pandemic period. In the pre-pandemic era, there were 1,000 (17.6%) patient deaths, whereas there were 160 deaths (21.3%, *P* = .01) during the pandemic. A total of 53 (42.1%) coronavirus disease 2019 (COVID-19) patients died having an HAI. Age and comorbidities increased the risk of death in patients with COVID-19 and an HAI. During the pandemic, Black patients with an HAI and COVID-19 were more likely to die than White patients with an HAI and COVID-19.

**Conclusions:**

In three Midwestern hospitals, patients with concurrent HAIs and COVID-19 were more likely to die if they were Black, elderly, and had certain chronic comorbidities.

## Introduction

Much has been written about severe acute respiratory syndrome coronavirus 2 (SARS-CoV-2) and its effects on mortality^1–4^ but relatively little on the effect of hospital-acquired infections (HAIs) on mortality among patients with SARS-CoV-2. In the initial phase of the pandemic, data reported to the National Healthcare Safety Network have demonstrated higher rates of central line-associated bloodstream infection, catheter-associated urinary tract infection, and ventilator-associated events (VAEs), compared to both pre-pandemic times and as a function of SARS-CoV-2 caseload, but these studies reported HAI incidence and did not comment on mortality.^5,6^

With this gap in understanding, we sought to use pre- and early-pandemic-era data to determine patient-level risk factors for mortality among patients with HAIs including urine, bloodstream, and respiratory infections. We used Fine–Gray competing risk models to investigate the impact of the pandemic on mortality in patients with an HAI in a network of St. Louis hospitals.

## Methods

### Data sources

This retrospective observational study was approved by the Washington University School of Medicine Institutional Review Board with a waiver of informed consent. Patient-level data were obtained for hospitalizations from January 1, 2017 through August 31, 2020 from the BJC HealthCare Clinical Data Repository for the three hospitals in the BJC System that treated the majority of coronavirus disease 2019 (COVID-19) patients, including one large quaternary care referral hospital and two community hospitals. The COVID-19 pandemic era defined to be from March 12, 2020 to the end of the study period. A randomly selected single admission was included for those with multiple admissions. All-cause mortality during the hospitalization was defined as the primary end point, with patients censored on September 30, 2020 if they were not discharged by then. Healthcare-associated infections (excluding *Clostridioides difficile* infection) were defined as those occurring ≥48 hours after admission and were based on positive urine, respiratory, and blood cultures, as defined in previous studies.^7^ The HAI date was defined as the first positive culture collection date. Patients could have more than one HAI during an admission, as defined by the Centers for Disease Control and Prevention (CDC’s) repeat infection time frame, that is, a 14-day time frame during which no new infections of the same type are reported.^8^

### Covariates

Inpatient stays at each facility of ≥48 hours duration were used to define monthly admission counts, total days admitted, and average length of stay (LOS). Demographics collected included sex, race, age, and payer; zip code was used to calculate zip-code level socioeconomic status measures, including the U.S. Census Bureau median household income and the CDC Social Vulnerability Index. Additional data included comorbidities defined using ICD-10-CM (International Classification of Diseases, Tenth Revision, Clinical Modification) diagnosis codes and the Elixhauser classification,^9,10^ body mass index (BMI), procedures/surgeries performed during admission (only those performed prior to an HAI) per ICD-10-PCS codes, admitting service defined as the first non-emergency department service per admission, inpatient medications, and ICU stay. Coronavirus disease 2019 was defined using laboratory results and ICD-10-CM diagnosis codes; a medical record review was performed for admissions with a COVID-19 ICD-10-CM diagnosis code without a positive laboratory result to confirm a clinical diagnosis of infection with SARS-CoV-2.

### Statistical analysis

Descriptive statistics were used to summarize the patients with categorical variables displayed as the number and percentage, and continuous variables as median with interquartile range. Group differences were investigated using the χ^2^ test (or variants thereof) for categorical and the Wilcoxon rank-sum test for continuous variables.

The primary analysis compared the estimates of unadjusted mortality patients with HAIs before and during the COVID-19 pandemic using a χ^2^ test.

In a further step, a Poisson interrupted times series was used to compare the unadjusted mortality trajectory for those with HAIs before and during the pandemic, using patient LOS as the denominator, and with the associated piece-wise model estimating differences between the two time periods.

The Aalen–Johansen estimator was plotted and group differences evaluated with the Gray’s test. In line with current thinking,^11^ supplementary analyses analyzed time to death and time to discharge within both a Fine–Gray competing risks framework and using cause-specific uni- and multivariable Cox proportional hazards (CPH) models for death and discharge separately. Time was measured from admission (“time 0”) to date of death, discharge, or administrative censoring (on 9/30/20). This necessarily introduced immortal time bias within the first 2 days, but this approach was considered more intuitive to understand than implicitly having to adjust for the 2-day time difference in analyses. In addition, we fitted a CPH model to estimate the mortality risk from having a COVID diagnosis, including inverse probability weights (IPWs) to adjust for baseline covariate imbalance between the two periods, using sandwich-type standard errors to account for potential intra-hospital correlation effects. We included all relevant baseline factors in the logistic regression model for the IPWs predicting the pre- (coded 0) and during-pandemic periods (coded 1), truncating at the 95% percentile (refer to the variables in Table S1). The most parsimonious models were determined by forward selection and backward deletion using the Akaike information criterion as the inclusion criterion. The proportional hazards assumption was confirmed both visually by plotting the Schoenfeld residuals and using the Grambsch–Therneau statistic.

Subgroup analyses of Black versus White race provided additional insights regarding potential discrepancies. A *post hoc* analysis was performed to investigate potential disparities between Black and White patients in more detail. We used 1:1 propensity score matching based on a nearest neighbor algorithm (package *MatchIt* in R) to identify an equal number of similar Black and White patients in terms of sex, age, BMI, health insurance (private or not), admitting hospital, median household income for the area in which the patient resides, comorbidities, and surgical procedures. We verified the matching process by comparing standardized mean differences and visual inspection of the propensity scores (details in the supplementary material Appendix). Following further stratification by household income (upper 50% quartile, lower 50% quartile), proportional hazards models were fitted and the predicted risk of death for Black and White patients compared during the pandemic.

All analyses were performed in R 3.6.1 (R Foundation for Statistical Computing, Vienna, Austria), with a *P*-value of less than .05 as statistically significant.

### Patient consent statement

This retrospective study was approved by the Washington University in St. Louis Institutional Review Board with a waiver of informed consent.

## Results

The final cohort included 6,447 HAI patients ≥18 years of age with admissions ≥48 hours across the three study hospitals from January 1, 2017 through August 31, 2020. Patients were predominantly White (67.9%), female (50.9% vs 46.1%, *P* = .02), having slightly lower BMI (28 vs 29, *P* = .001), and more having private insurance (50.6% vs 45.7%, *P* = .01) in the pre-pandemic compared to the pandemic period (Table 1). Baseline characteristics were broadly comparable between the two periods, but with slightly higher proportions of Black patients hospitalized during the pandemic (27.6% pre vs 34.1% during the pandemic). In the pre-pandemic era, there were 1,000 (17.6%) patient deaths, whereas during the SARS-CoV-2 era, there were 160 deaths (21.3%, *P* = .01). Among COVID-19 patients who developed an HAI, 53 (42.1%) died during hospitalization.

**Table 1.** Characteristics of HAI patients, stratified by pre and during pandemic

Characteristic N (%)/median [IQR]	Total cohort	Pre-pandemic	During pandemic	*P*-value^a^	During pandemic HAI and COVID diagnosis
Number of patients	6447	5697	750	–	126
**Primary end point:** ^a^					
Death (all-cause)	1160 (18.0)	1000 (17.6)	160 (21.3)	.01	53 (42.1)
Length of stay (days)	19 [11, 31]	19 [11, 31]	21 [13, 35]	<.001	
Sex: female	3246 (50.3)	2900 (50.9)	346 (46.1)	.02	49 (38.9)
Age	65 [54, 74]	65 [54, 74]	64 [53, 73]	.09	66 [56, 73]
Race				<.001	
Black	1814 (28.1)	1557 (27.3)	257 (34.3)		84 (66.7)
White	4377 (67.9)	3905 (68.5)	472 (62.9)		36 (28.6)
Other	256 (4.0)	235 (4.1)	21 (2.8)		6 (4.8)
BMI	28 [23, 34]	28 [23, 33]	29 [25, 34]	.001	32 [27, 38]
Hospital				.3	
1	4651 (72.1)	4107 (72.1)	552 (73.6)		64 (50.8)
2	743 (11.5)	648 (11.4)	91 (12.1)		41 (32.5)
3	1053 (16.3)	942 (16.5)	107 (14.3)		21 (16.7)
Private insurance	3227 (50.1)	2882 (50.6)	343 (45.7)	.01	53 (42.1)

HAI, hospital-acquired infection; IQR, interquartile range.

a Comparing the pre- and during-pandemic groups.

**Figure 1.**
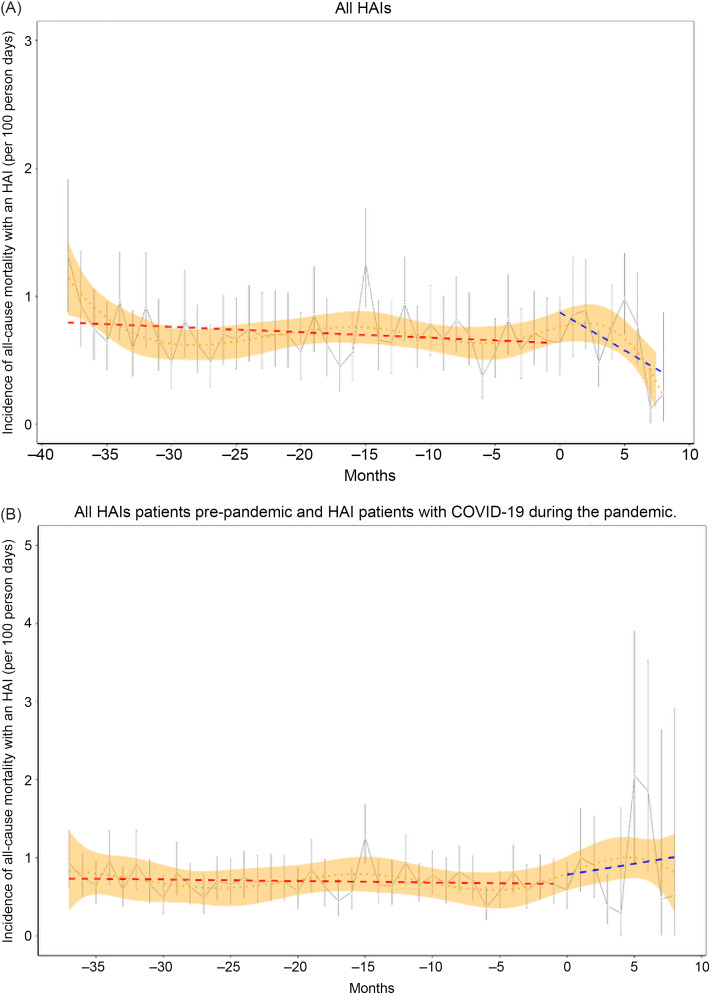
Interrupted time series comparing unadjusted monthly all-cause mortality among A. those with an HAI (gray, with point-wise 95% confidence intervals) before the pandemic (linear fit, red dashed) and during the pandemic (linear fit, blue dashed) and B. those with HAI and COVID during the pandemic; time 0 is March 12, 2020; restricted cubic spline smoother with 6 knots indicates the trajectory over the whole period (orange dotted, with 95% confidence interval shaded).

Figure 1A shows an interrupted time series analysis comparing the trajectory of unadjusted monthly all-cause mortality for patients with an HAI pre-pandemic and during the pandemic. There was a decrease in mortality in the last 2 months of the pandemic period (after an initial increase in the early pandemic), but otherwise mortality was fairly constant. Mortality for those with an HAI increased slightly during the pandemic for COVID-19 patients (Fig. 1B, *P*-value = .1 for an increase in slope). Cox proportional hazards models comparing all-cause mortality in patients with an HAI prior to and during the pandemic confirmed older age as a risk factor (Table S1). Of note, for those with a COVID diagnosis and an HAI, there was a nonlinear effect in patient age with risk increasing for people of age >65 years (adjusted hazard ratio (aHR) 3.0, 95% confidence interval (CI) [2.3, 4.0], *P* < .001), and for those with chronic lung disease (aHR 1.9, 95% CI [1.2, 2.2], *P* < .001) and elevated BMI (aHR 1.6 per 5-point increase, 95% CI [1.4, 1.8], *P* < .001). Having a COVID diagnosis marginally increased the risk of all-cause death for those with an HAI in inverse probability weighted and adjusted CPH models (aHR 1.7 [1.0, 2.4], *P* = .08, Table 2)).

**Table 2.** Estimated effect of having a COVID diagnosis and an HAI on all-cause mortality from fitted Cox proportional hazards models (N = 6389 patients)

End point: death (all-cause)	Univariable		Multivariable^a^	
	HR (95% CI)	*P*-value	aHR (95% CI)	*P*-value
COVID diagnosis (unweighted)	1.7 (1.2, 2.5)	.002	1.6 (1.1, 2.3)	.02
COVID diagnosis (weighted)	1.7 (1.1, 2.7)	.01	1.5 (1.0, 2.4)	.08

HAI, hospital-acquired infection; HR, hazard ratio; aHR, adjusted hazard ratio.

a Adjusted for sex, age, BMI, race, hospital, and private insurance (yes/no).

Table S3 demonstrates the percentages of HAI by type pre and during the pandemic. The percentage of respiratory HAIs increased during the pandemic but without a concomitant increase in mortality. Having a COVID diagnosis increased mortality rates across all HAIs. Urine HAIs were the most common, followed by respiratory and then blood.

### Mortality and race

**Figure 2.**
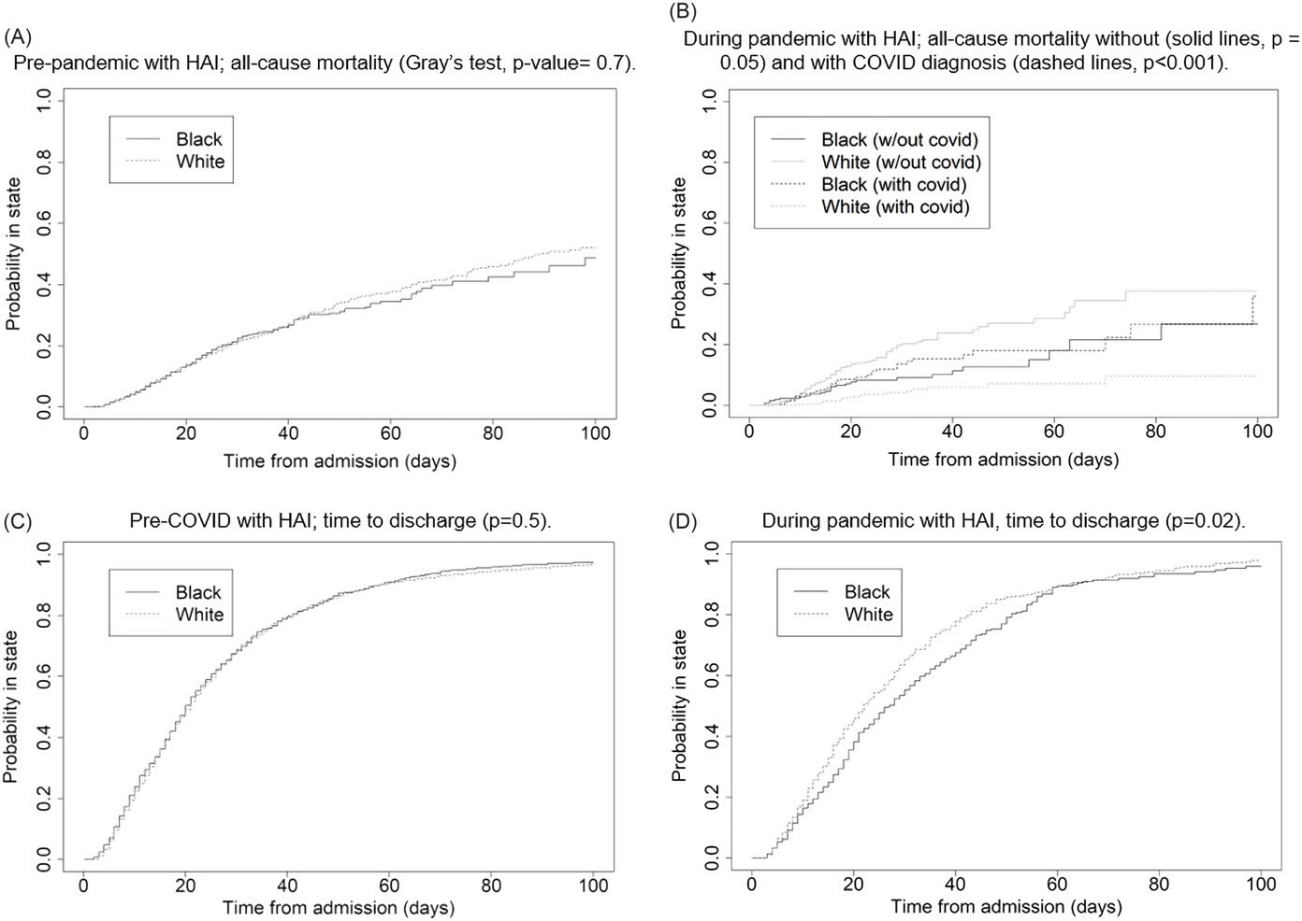
Aalen–Johansen estimator for the competing risk of death (with and without COVID), and discharge while having an HAI, stratified by pre and during pandemic, and race.

**Figure 3.**
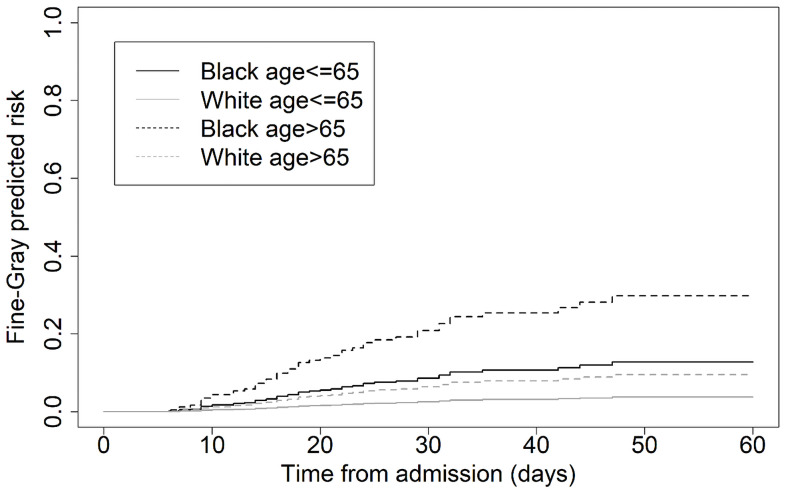
Estimated predicted risk profiles from the Fine–Gray sub-distribution proportional hazards model for those dying with both an HAI and COVID infection (N = 729 patients), stratified by race and age.

A subgroup analysis of Black and White patients suggests that prior to the pandemic, patients with an HAI died (and were discharged) at approximately the same rate, regardless of race (Fig. 2 panels A and C, respectively). However, during the pandemic, Black patients with an HAI and COVID were more likely to die than White patients with an HAI and COVID (panels B and D, respectively). Furthermore, the predictive distribution from the Fine–Gray proportional hazards model suggested that this difference was almost exclusively among patients over the age of 65, albeit with only in total 31 deaths (22 Black/9 White) in this subgroup (HR for Black patients 4.9, 95% confidence interval [2.3, 10.5], *P* < .001, refer to Fig. 3).

In a further subgroup analysis with patients during the pandemic, selecting matched pairs of Black and White patients based on propensity scores (using the covariates in Table S1) indicated that discrepancies were more pronounced in a subgroup of patients with lower median household income (refer to supplementary material, Figure SG1, and the Appendix). However, given the low number of patients and deaths included in these analyses, especially those with a COVID diagnosis, these results are indicative only.

## Discussion

In this study, we show that patients with a COVID-19 diagnosis and an HAI during their hospitalization were marginally more likely to die than patients without COVID-19 and an HAI in the pandemic. One previous study showed an increase in HAIs and mortality during the pandemic,^12^ but none have specifically studied the interaction between COVID-19, HAIs, and mortality. In a study on VAEs, pandemic-era patients with VAEs were equally likely to die whether or not they had COVID-19.^13^ In a small study of ICU patients, HAIs were not associated with an increase in mortality in COVID-19 patients.^14^ There have been a few studies showing an association between COVID, HAIs, and increased risk of death,^15–17^ including one study where this association only held true if the HAI caused septic shock.^18^

Severe acute respiratory syndrome coronavirus 2 affects people of color more than White patients including higher mortality rates.^19^ Though some studies have shown Black patients are not more likely to die after adjusting for sociodemographic factors and comorbidities,^20^ we know that Black patients, because of structural racism, are more likely to be poor and have chronic health conditions, which, in effect, means that structural racism has led them to be at higher risk of dying from COVID-19. Our study suggests that Black patients, particularly elderly patients and those with lower household incomes, are more likely to die with COVID-19 and an HAI compared to White patients.

Our study is limited by its regional nature, with all three hospitals located in the St. Louis metropolitan area, which may not be representative of patient populations and risk factors for HAIs and mortality across the state, country, and globe. In addition, as these are observational data, we cannot be certain that the results are a direct result of the pandemic and not some other confounding factor. In particular, the assumption of “no unmeasured confounding” in our weighted analyses was a strong one; the inpatient population was known to be different during the pandemic (e.g. fewer routine surgeries), and the influence of increased general hygiene measures (e.g., masking, cohorting, etc.) cannot be estimated. Our study is limited by the amount of data available, which reduces our statistical power, and may mean that some of the null results we found are due to lack of power rather than lack of true effect. This can be explored further in future studies with larger cohort sizes.

Our study is strengthened by the rigorous analytical method utilized, including Fine–Gray competing risk models, multivariable logistic regression, and interrupted time series.

In conclusion, the patients with HAIs and COVID-19 were more likely to die if they were Black, elderly, and had lower income compared to White patients of a similar age and income bracket.

## Supporting information

Atkinson et al. supplementary material Atkinson et al. supplementary material

## Supplementary material

For supplementary material accompanying this paper visit http://doi.org/10.1017/ash.2023.409. click here to view supplementary material
