# Supplementary material for: Impact of the Severe acute respiratory syndrome coronavirus 2 pandemic on mortality associated with healthcare-associated infections
Source: Antimicrob Steward Healthc Epidemiol. 2023 Aug 29;3(1):e142. doi: 10.1017/ash.2023.409 (PMC10523544; doi:10.1017/ash.2023.409)

Supplementary Material

Subgroup analysis

Rationale: Propensity score match on the subgroup of patients during the pandemic in the lower and upper half of the household income distribution, then repeating the Fine-Gray analysis from Figure 3 for those with a COVID diagnosis and HAI.

Figure SG1: Propensity score 1:1 matched analysis stratified by median annual Household income (HHI)

A. Upper 50% HHI (>$51k) matched 2 x 63 patients B. Lower 50% (<$51k) 2 x 172 matched patients

(N=15 deaths with COVID, p=0.3). (N=28 deaths, p=0.006); note - median HH income 6k lower in “Black” and 16% less with private insurance.


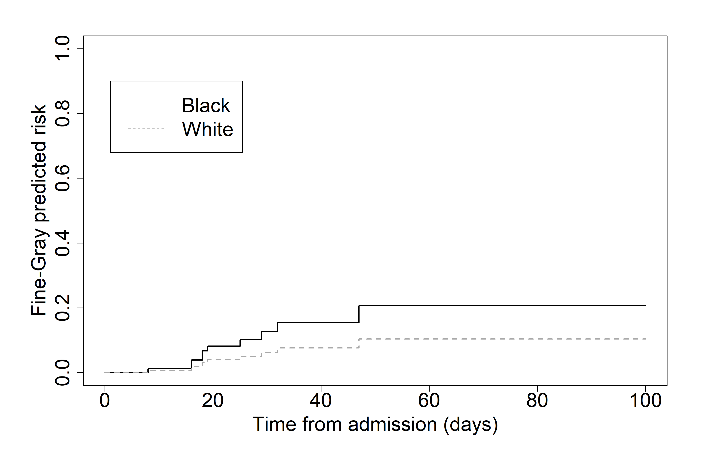

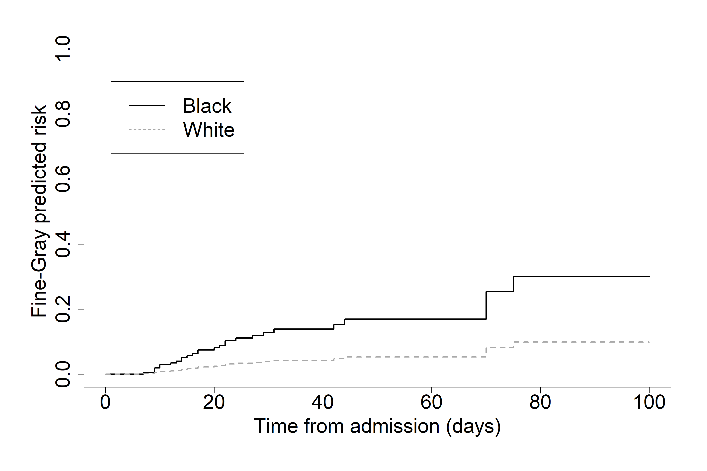


Table S1: Estimates from cause-specific Cox proportional hazards models for those with HAIs taking all-cause mortality as dependent variable prior to the pandemic (1), during the pandemic (2a), and for those patients with a COVID diagnosis (2c). Risk factors consistent across all 3 analyses shown in **bold**, with COVID specific trends in *italics*; HR>1 indicates increased risk of death.

| Endpoint:  Death (all-cause) | **1. Pre-pandemic**  (N=5697 patients, 1000 deaths) | | | | **2. During pandemic** | | | | | | | |
| --- | --- | --- | --- | --- | --- | --- | --- | --- | --- | --- | --- | --- |
|  |  |  |  |  | a.) All  (N=750 patients, 160 deaths) | | | | b.) With a COVID diagnosis  (N=126 patients, 53 deaths) | | | |
| Analysis | Univariable | | Multivariable  (N=5644 patients) | | Univariable | | Multivariable  (N=745 records) | | Univariable |  | Multivariable  (N=126 records) |  |
| Risk factor | HR (95% CI) | p-value | aHR (95% CI) | p-value | HR (95% CI) | p-value | aHR (95% CI) | p-value | HR (95% CI) | p-value | aHR (95% CI) | p-value |
| Sex  Female  Male | 1 (reference)  1.2 (1.1, 1.4) | 0.002 | 1 (reference)  1.2 (1.1, 1.3) | <0.001 | - | NS | - | - | 1 (reference)  1.4 (1.1, 1.8) | 0.003 | - | - |
| Age (10 year steps)  **Age (>65 years)** | 1.2 (1.2, 1.3)  **1.7 (1.5, 1.9)** | <0.001  **<0.001** | -  **1.5 (1.4, 1.6)** | nE  <0.001 | 1.2 (1.1, 1.3)  **1.7 (1.5, 1.9)** | <0.001  **<0.001** | -  **1.5 (1.3, 1.7)** | nE  **<0.001** | 1.2 (1.0, 1.5)  **1.8 (1.2, 2.9)** | 0.03  **0.006** | -  **3.0 (2.3, 4.0)** | nE  **<0.001** |
| BMI (5 point steps) | 0.9 (0.8, 0.9) | <0.001 | 0.9 (0.8, 0.9) | <0.001 | 0.9 (0.8, 0.9) | <0.001 | - | NS | 1.2 (1.0, 1.4) | 0.1 | 1.6 (1.4, 1.8) | <0.001 |
| Race  Black  White  Other | 1 (reference)  -  1.4 (1.0, 1.8) | NS  0.03 | 1 (reference)  -  - | NS  NS | 1 (reference)  -  1.7 (1.1, 2.9) | NS  0.03 | -  - | NS  NS | -  0.6 (0.5, 0.6) | NS  <0.001 | -  - | -  NS |
| Hospital  1  2  3 | 1 (reference)  **1.5 (1.2, 1.8)**  1.5 (1.2, 1.8) | **<0.001**  <0.001 | 1 (reference)  **1.3 (1.3, 1.6)**  1.4 (1.3, 1.4) | **<0.001**  <0.001 | 1 (reference)  **1.8 (1.8, 1.8)**  1.3 (1.3, 1.3) | **<0.001**  <0.001 | 1 (reference)  **1.6 (1.5, 1.7)**  1.5 (1.5, 1.6) | **<0.001**  <0.001 | 1 (reference)  **0.7 (0.7, 0.7)**  0.9 (0.9, 0.9) | **<0.001**  <0.001 | 1 (reference)**0.6 (0.5, 0.8)**  - | **0.002**  NS |
| Private insurance | - | NS | - | - | 1.1 (1.0, 1.3) | 0.01 | - | NS | - | NS | - | - |
| Comorbidities^1^ |  |  |  |  |  |  |  |  |  |  |  |  |
| Pulmonary circulation disease | - | NS | - | - | - | NS | - | - | - | NS | - | - |
| Hypertension | 1.2 (1.0, 1.5) | 0.02 | - | NS | - | NS | - | - | - | NS | - | - |
| Chronic pulmonary disease | - | NS | - | - | 1.2 (1.0, 1.6) | 0.05 | 1.2 (1.2, 1.3) | <0.001 | 1.7 (1.4, 2.1) | <0.001 | 1.9 (1.7, 2.2) | <0.001 |
| Chronic kidney disease | 1.4 (1.2, 1.6) | <0.001 | 1.1 (1.1, 1.1) | <0.001 | - | NS | - | - | - | NS | - | - |
| Coagulopathy | 1.5 (1.5, 1.6) | <0.001 | 1.6 (1.5, 1.6) | <0.001 | 1.8 (1.5, 2.1) | <0.001 | 1.8 (1.5, 2.0) | <0.001 | - | NS | - | - |
| Obesity | 0.8 (0.8, 0.9) | <0.001 | - | NS | 0.6 (0.5, 0.7) | <0.001 | 0.5 (0.4. 0.6) | <0.001 | - | NS | - | - |
| Deficiency anemias | 1.2 (1.1, 1.3)^!^ | 0.01 | 0.9 (0.9, 1.0) | 0.04 | 0.8 (0.8, 0.8) | <0.001 | 0.7 (0.6, 0.8) | <0.001 | - | NS | - | - |
| Diabetes | 1.1 (1.0, 1.3) | 0.02 | - | NS | - | NS | - | - | 0.5 (0.4, 0.7) | <0.001 | 0.5 (0.4, 0.8) | <0.001 |
| Immuno-suppression |  |  |  |  |  |  |  |  |  |  |  |  |
| Lymphoma | - | NS | - | - | 0.4 (0.3, 0.5) | <0.001 | 0.5 (0.4, 0.6) | <0.001 | *-* | NS | - | *-* |
| Metastatic cancer | 1.8 (1.6, 2.2) | <0.001 | 1.8 (1.6, 2.1) | <0.001 | - | NS | - | - | - | NS | - | - |
| Oncologic procedure | 0.8 (0.7, 0.9)^!^ | <0.001 | - | NS | 0.8 (0.7, 0.9) | 0.01 | 0.4 (0.4, 0.5) | <0.001 | - | nE | - | - |
| Leukemia diagnosis | 0.7 (0.7, 0.8)^!^ | <0.001 | 0.8 (0.7, 0.9) | <0.001 | 1.7 (1.4, 2.0) | <0.001 | 2.8 (2.6, 3.0) | 0.001 | - | nE | - | - |
| Transplant | - | NS | - | - | - | nE | - | - | - | nE | - | - |
| Procedures (before HAI)^1^ |  |  |  |  |  |  |  |  |  |  |  |  |
| Renal procedure | 1.5 (1.1, 2.1) | 0.01 | 1.2 (1.4, 1.7) | <0.001 | - | NS | - | - | - | NS | - | - |
| Cardiac procedure | - | NS | - | - | - | NS | - | - | *1.4 (1.1, 1.7)* | *0.006* | - | NS |
| Catheter | 1.1 (1.0, 1.3) | 0.06 | - | - | - | NS | - | - | 0.8 (0.6, 1.0) | 0.04 | 0.7 (0.5, 0.9) | 0.009 |
| Neuro | 0.8 (0.7, 0.9) | <0.001 | 0.9 (0.9, 0.9) | <0.001 | 0.4 (0.3, 0.5) | <0.001 | 0.4 (0.4, 0.5) | <0.001 | - | NS | - | - |
| Orthopedic | 0.8 (0.7, 0.9) | 0.003 | 0.9 (0.8, 1.0) | 0.05 | 0.6 (0.3, 0.9) | 0.009 | 0.7 (0.5, 1.0) | 0.03 | - | nE | - | - |
| Surgery | **0.8 (0.8, 0.9)^!^** | **<0.001** | **0.8 (0.7, 0.9)** | **<0.001** | **0.5 (0.4, 0.7)** | **<0.001** | **0.5 (0.4, 0.7)** | **<0.001** | **-** | NS | **-** | **-** |
| Thoracic | 0.8 (0.7, 0.9) | 0.004 | 0.8 (0.7, 0.9) | <0.001 | - | NS | - | - | - | - | - | - |
| Collagen vascular disease | - | NS | - | - | - | NS | - | - | *0.5 (0.4, 0.7)* | *<0.001* | - | NS |

NS – not significant at the 10% level; nE not estimated (collinear with another variable or too few patients); ^1^not shown (not significant at the 10% level in univariable analyses): comorbidities -rheumatoid arthritis, solid tumor without metastasis, vascular procedure; ^!^Grambsch-Thernau test for proportional hazards, p-value < 0.05 (ie rejecting the null hypothesis of proportionality).

Table S2: Patient characteristics for the total cohort at baseline, and stratified by pre- and during pandemic (*long version of Table 1*)

| Characteristic  N (%) / median [IQR] | Total cohort | Pre-pandemic | During pandemic | p-value^1^ | During pandemic  HAI and COVID diagnosis |
| --- | --- | --- | --- | --- | --- |
| Number of patients | 6447 | 5697 | 750 | - | 126 |
| **Primary Endpoint:**  Death (all-cause) | 1160 (18.0) | 1000 (17.6) | 160 (21.3) | 0.01 | 53 (42.1) |
| Length of stay (days) | 19.00 [11, 31] | 19 [11, 31] | 21 [13, 35] | <0.001 | 28 [19, 41] |
| Sex – female | 3246 (50.3) | 2900 (50.9) | 346 (46.1) | 0.02 | 49 (38.9) |
| Age | 65 [54, 74] | 65 [54, 74] | 64 [53, 73] | 0.09 | 66 [56, 73] |
| Race  Black  White  Other | 1814 (28.1)  4377 (67.9)  256 (4.0)) | 1557 (27.3)  3905 (68.5)  235 (4.1) | 257 (34.3)  472 (62.9)  21 (2.8) | <0.001 | 84 (66.7)  36 (28.6)  6 (4.8) |
| BMI | 28 [23, 34] | 28 [23, 33] | 29 [25, 34] | 0.001 | 32 [27, 38] |
| Hospital  1  2  3 | 4651 (72.1)  743 (11.5)  1053 (16.3) | 4100 (72.0)  651 (11.4)  946 (16.6) | 551 (73.5)  92 (12.3)  107 (14.3) | 0.2 | 64 (50.8)  41 (32.5)  21 (16.7) |
| Private insurance | 3227 ( 50.1) | 2884 (50.6) | 343 (45.7) | 0.01 | 53 (42.1) |
| Comorbidities |  |  |  |  |  |
| Pulmonary circulation disease | 393 ( 6.1) | 334 (5.9) | 59 (7.9) | 0.09 | 12 (9.5) |
| Hypertension | 4971 (77.1) | 4368 (76.7) | 603 (80.4) | 0.07 | 119 (94.4) |
| Chronic pulmonary disease | 2145 (33.3) | 1877 (32.9) | 268 (35.7) | 0.3 | 46 (36.5) |
| Chronic kidney disease | 2189 (34.0) | 1942 (34.1) | 247 (32.9) | 0.7 | 53 (42.1) |
| Coagulopathy | 2107 (32.7) | 1796 (31.5) | 311 (41.5) | <0.001 | 66 (52.4) |
| Obesity | 1622 (25.2) | 1407 (24.7) | 215 (28.7) | 0.06 | 55 (43.7) |
| Deficiency anemias | 2400 (37.2) | 2093 (36.7) | 307 (40.9) | 0.07 | 77 (61.1) |
| Diabetes | 2475 (38.4) | 2172 (38.1) | 303 (40.4) | 0.4 | 75 (59.5) |
| Immuno-suppression |  |  |  |  |  |
| Lymphoma | 352 (5.5) | 318 (5.6) | 34 (4.5) | 0.4 | 1 (0.8) |
| Metastatic cancer | 486 (7.5) | 432 (7.6) | 54 (7.2) | 0.8 | 3 (2.4) |
| Oncologic procedure | 385 (6.0) | 359 (6.3) | 26 (3.5) | 0.003 | 0 (0.0) |
| Leukemia diagnosis | 369 (5.7) | 333 (5.8) | 36 (4.8) | 0.5 | 1 (0.8) |
| Transplant | 78 ( 1.2) | 75 (1.3) | 3 (0.4) | 0.09 | 0 (0.0) |
| Procedures* |  |  |  |  |  |
| Renal procedure | 645 (10.0) | 547 (9.6) | 98 (13.1) | 0.04 | 40 (31.7) |
| Cardiac procedure | 1195 (18.5) | 1066 (18.7) | 129 (17.2) | 0.3 | 12 (9.5) |
| Catheter | 2205 (34.2) | 1896 (33.3) | 309 (41.2) | <0.001 | 81 (64.3) |
| Neuro | 926 (14.4) | 804 (14.1) | 122 (16.3) | 0.1 | 6 (4.8) |
| Orthopedic | 262 (4.1) | 222 (3.9) | 40 (5.3) | 0.08 | 0 (0.0) |
| Surgery | 1298 (20.1) | 1170 (20.5) | 128 (17.1) | 0.03 | 9 (7.1) |
| Thoracic | 1152 (17.9) | 1011 (17.7) | 141 (18.8) | 0.5 | 21 (16.7) |
| Vascular | 1067 (16.6) | 925 (16.2) | 142 (18.9) | 0.07 | 23 (18.3) |

^1^comparing the pre- and during pandemic groups;*procedures before the HAI.

Appendix: Propensity score matching of Black and white patients during COVID.

Upper Quartile of the HHI distribution

| \| | Control | Treated |
| --- | --- | --- |
| \|All | 298 | 63 |
| \|Matched | 63 | 63 |
| \|Unmatched | 235 | 0 |
| \|Discarded | 0 | 0 |
|  |  |  |

Black white

discharge 50 51

**death (w/out covid) 6 9**

death (with covid) 7 3

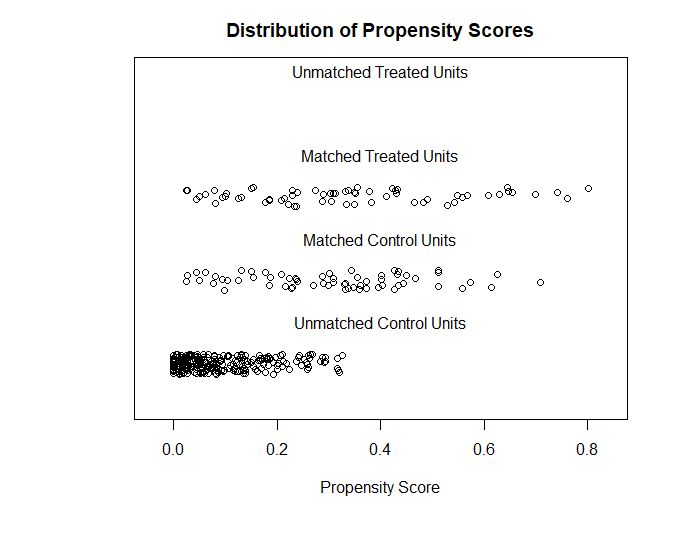


Lower Quartile of the HHI distribution

| \| | Control | Treated |
| --- | --- | --- |
| \|All | 189 | 172 |
| \|Matched | 172 | 172 |
| \|Unmatched | 17 | 0 |
| \|Discarded | 0 | 0 |

Black white

discharge 131 142

death (w/out covid) 19 24

**death (with covid) 22 6**

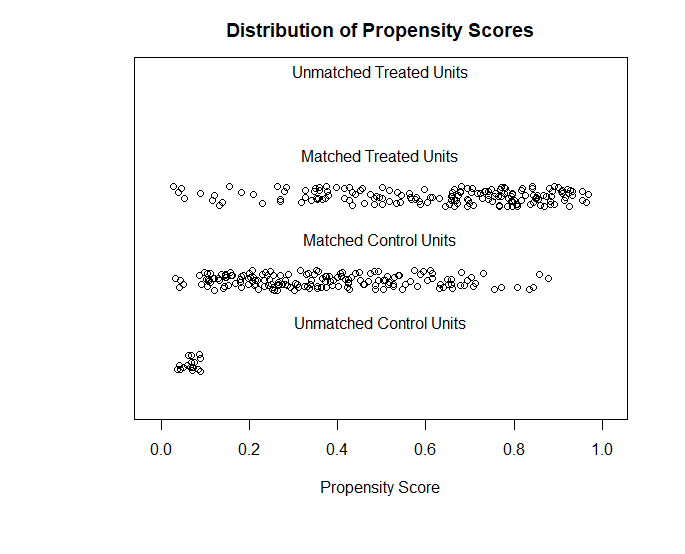

Supplement: Atkinson et al. supplementary material [file S2732494X23004096sup001.docx]
